# Prevalence and factors associated with early antenatal care visit in Ghana: recent evidence from the 2022 Ghana Demographic and Health Survey

**DOI:** 10.1016/j.xagr.2026.100605

**Published:** 2026-01-10

**Authors:** Befkad Derese Tilahun, Mulat Ayele, Eyob Shitie Lake, Bogale Molla, Gizachew Yilak, Molla Azmeraw, Tegene Atamenta Kitaw

**Affiliations:** 1 Departments of Nursing (Tilahun, Molla, Yilak, Azmeraw, and Kitaw); 2 Midwifery (Ayele and Lake), College of Health Science, Woldia University, Woldia, Ethiopia

**Keywords:** Antenatal care, determinants, Ghana, prevalence

## Abstract

**Introduction:**

Antenatal care (ANC) is a critical component of the maternal and child health continuum, and early initiation is essential to achieving optimal outcomes for both mothers and infants. Although the World Health Organization recommends initiating ANC within the first 12 weeks of gestation, many women in developing countries, including Ghana, do not meet this guideline.

**Methods:**

This study examined the prevalence of early ANC initiation and its associated factors among Ghanaian women using a weighted sample of 5,075 respondents. Data were analyzed with STATA/SE version 17, applying descriptive statistics and binary logistic regression to identify predictors of early initiation, with variables significant at *p*≤0.05 retained in the final model.

**Results:**

Overall, 65.1% of women initiated ANC within the recommended period. Urban residence (adjusted odds ratio [AOR], 1.87; 95% CI, 1.95–2.32), tertiary or higher maternal education (AOR, 2.53; 95% CI, 1.72–3.73), and higher socioeconomic status (AOR, 1.33; 95% CI, 1.07–1.66) were positively associated with early initiation, whereas higher parity (≥3 children) was negatively associated (AOR, 0.59; 95% CI, 0.46–0.76).

**Conclusion:**

These findings highlight that socioeconomic and demographic factors significantly influence ANC timing, underscoring the need for interventions targeting rural women, those with limited education, and economically disadvantaged groups to promote timely initiation of ANC.

**AJOG Global Reports at a Glance**

**Why was this study conducted?**

This study aimed to determine the prevalence of early antenatal care (ANC) initiation in Ghana using the 2022 Ghana Demographic and Health Survey dataset and to identify key demographic and socioeconomic factors associated with early ANC booking.

**Key findings**

Overall, 65.1% of pregnant women initiated ANC within the first 12 weeks of gestation. Early ANC was more likely among women in urban areas, with higher education, and from wealthier households. Women with higher parity (≥3 children) had significantly lower odds of early ANC initiation.

**What does this add to what known?**

Our study provides the most recent nationally representative evidence on early ANC in Ghana; demonstrates that education, residence, wealth, and parity remain central predictors of early ANC; and highlights priority groups rural, less-educated, and high-parity women for targeted maternal health interventions.

## Background

Antenatal care (ANC) serves as a fundamental pillar of the maternal and child healthcare continuum.^1^ This specialized care that is provided by trained health professionals focuses on keeping pregnant women and their developing fetuses healthy throughout pregnancy. ANC includes various elements, such as identifying risks, preventing and managing complications related to pregnancy, and implementing health education and promotion strategies.^2^ In line with global best practices, the World Health Organization’s (WHO) focused ANC (FANC) model emphasizes the importance of initiating ANC during the first trimester of pregnancy (before 12 weeks of gestation) for optimal maternal and fetal outcomes.^3^

Globally, approximately 515,000 women lose their lives due to complications related to pregnancy each year.^4^ In addition, more than 30 million women in developing countries experience severe obstetrical complications annually.^5^ Ghana’s maternal mortality ratio (MMR) decreased substantially from 760 deaths per 100,000 live births in 1990 to 319 per 100,000 in 2015.^6^ However, progress has slowed since then, with the ratio remaining largely unchanged at approximately 308 deaths per 100,000 live births in 2022.^7^

Early initiation of ANC plays a paramount role in enhancing maternal health as it provides an opportunity for the early screening, treatment, and referral of pregnancy complications.^8^ In sub-Saharan Africa, where complications during pregnancy lead to higher maternal mortality rates than in other areas, the delay and lack of access to ANC services are significant contributing factors.^9^

Delayed initiation of ANC is linked to negative pregnancy outcomes, including perinatal mortality, stillbirth, and early neonatal death.^10^ This delay increases the risks of maternal mortality during pregnancy and delivery, along with both acute and chronic health issues in mothers.^11^ It is important to note that approximately 25% of maternal deaths occur during the antenatal period, primarily due to preventable or treatable conditions, such as preeclampsia, eclampsia, and antepartum hemorrhage, which are manageable with timely ANC interventions.^12^

Several studies highlight important factors that affect the timing of early ANC registration, including maternal education,^13^, ^14^, ^15^ maternal age,^16^ parity,^17^^,^^18^ occupational status,^19^^,^^20^ wealth status,^13^^,^^21^ husband education,^14^^,^^22^ distance from health facility,^22^^,^^23^ marital status,^24^ residence,^25^ and religion.^24^

By 2030, one of the main goals of the Ending Preventable Maternal Mortality initiative is to bring the global MMR down to below 70 deaths per 100,000 live births. Furthermore, countries are anticipated to reduce their MMRs by at least two-thirds from their 2010 starting points, ensuring that no nation has an MMR exceeding 140 maternal deaths per 100,000 live births by 2030.^26^

In 2002, Ghana’s government implemented the WHO’s FANC initiative to reduce the alarmingly high maternal mortality rate and enhance the availability, standards, and consistency of prenatal services for pregnant women.^27^ In addition, Ghana has made significant progress in reducing maternal and child mortalities by half through the implementation of a free maternal healthcare service package. However, maternal mortality remains high at 412 per 100,000 live births, whereas child mortality stands at 17 per 1000 live births.^28^

Despite 98% of pregnant women in Ghana enrolling in ANC, early initiation remains a challenge, with limited research on its influencing factors. Although early ANC is crucial for maternal and child health, no study in Ghana has explored its determinants. To address this gap, this study aimed to evaluate the prevalence and key determinants of early ANC booking in Ghana, providing evidence to enhance maternal healthcare strategies.

## Materials and methods

### Study setting, study period, and data source

The 2022 Ghana Demographic and Health Survey (GDHS) was used as the data source for this study. The GDHS is part of the Demographic and Health Survey (DHS) program, which collects health and demographic information on women, men, and children worldwide. Since its inception, the DHS has been conducted in more than 90 low- and middle-income countries, with more than 350 surveys completed.

After the initial survey in 1988, the 2022 GDHS is Ghana’s ninth regular DHS. Structured questionnaires were used to collect data, and a cross-sectional design and multistage sampling technique were used. A nationally representative sample of 15,014 women between the ages of 15 and 49 years participated in the study.

The GDHS offers detailed data on the background characteristics of respondents, maternal healthcare, fertility, marriage and sexual activity, child feeding patterns, maternal and child nutritional status, and death rates for adults and children.

### Data extraction, population

We were given permission to acquire the 2022 GDHS data from the DHS program after submitting a legitimate request and receiving a letter of authorization. Data extraction was performed to identify pregnant mothers, resulting in a total of 5075 cases. Therefore, the final weighted sample size was 5075.

The timeframe for data extraction was from February 2, 2025, to March 1, 2025. The study population included all pregnant women from the chosen enumeration districts throughout the same period, whereas the source population consisted of all pregnant women during the 5 years before the survey.

### Sampling methods

The 2022 GDHS used a robust stratified 2-stage sampling methodology to ensure national representativeness. Initially, all regions were stratified into urban and rural categories, creating 21 distinct sampling strata that accounted for geographic and residential variations. In the first sampling stage, 305 enumeration areas (EAs) were carefully selected through probability proportional to size sampling, involving 93 urban EAs and 212 rural EAs to appropriately reflect population distribution patterns. The second stage involved systematically selecting a consistent sample of 30 households per EA with equal probability from updated household listings, a methodological choice that balanced fieldwork feasibility with statistical precision while ensuring that each household had an equal chance of selection. Further details on the sampling procedure can be found in the 2022 GDHS report.^29^ For this study, a total of 5075 weighted pregnant mothers were included.

### Inclusion and exclusion criteria

All pregnant women between the ages of 15 and 49 years whose gestational age was known at the time of their first ANC appointment were included in the study. Women whose gestational age was documented at delivery or pregnancy termination were also included, as were those who did not receive ANC during their pregnancy. However, the study did not include women whose gestational age at the time of their first ANC appointment was unclear or unrecorded.

### Study variables

#### Outcome/dependent variable

The outcome variable of this study was early ANC initiation. Early ANC initiation is defined as the initiation of ANC services before the first 12 weeks of pregnancy.^30^

#### Independent variables

The independent variables included age, religion, educational level, employment or current working status, marital status, the total number of children ever born (parity), media exposure (reading newspapers or magazines, listening to the radio, or watching television), health insurance coverage, and husband’s education. The contextual variables included the sex of the household head, wealth index, place of residence, and region of residence. We identified and obtained the variables by their inclusion in the DHS data and by conducting an extensive review of relevant literature (Table 1).

**Table 1:** List of explanatory variables for the assessment of early ANC booking in Ghana

Variable	Descriptions
Woman’s age	The ages of pregnant mothers were categorized as 15–24, 25–34, and 35–49 y.
Residence	Women’s residence place was urban or rural.
Mother’s educational level	Women’s level of education was classified as no education, primary, secondary, or higher.
Wealth index	It was categorized as poor, middle, and rich.
Marital status	Not married or married.
Parity	The number of children ever born, including the current pregnancy, was categorized as ≤2 and ≥3.
Sex of the household head	Head of the household was categorized as male or female.
Household size	Household size was classified as 1–4, 5–9, and ≥10.
Distance to the health facility	Big problem. Not a big problem.
Age of the household	<35, 35–44, 45–54, 55–64, or >65 y
Birth order	First, second, third, or fourth.
Parental educational level	No education, primary, secondary, or higher.
Media exposer	No or yes.

*ANC*, antenatal care.

#### Operational definition

The variable for early ANC initiation is defined as follows:

• Yes (“0”): The woman initiated ANC before 12 weeks of gestation. This indicates adherence to recommended early prenatal care guidelines.
• No (“1”): The woman initiated ANC after 12 weeks of gestation, which is considered delayed and suboptimal for maternal and fetal health monitoring.^30^^,^^31^

#### Data processing and analysis

The analysis used Stata (version 17; StataCorp, College Station, TX) to process data from the 2022 GDHS women’s dataset. Data were processed and refined through coding, cleaning, and editing procedures. The missing values were identified using listing and sorting techniques. Descriptive statistics, including weighted frequencies and percentages, were computed to summarize the key variables. To ensure nationally representative estimates, adjustments were applied for sampling weight (v005), primary sampling unit (v023), and stratification (v021).

Logistic regression was used to explore determinants of early ANC initiation. At the univariate stage, frequencies and percentages were examined for all variables. Subsequently, crude and adjusted binary logistic regression models were developed to evaluate associations between independent variables and early ANC attendance. Statistical significance was determined using a 95% confidence interval and a *P* value threshold of 5%, with results presented for both unadjusted and multivariate-adjusted analyses.

## Results

### Sociodemographic and household characteristics

The study population predominantly consisted of young mothers, with nearly half of the population (47.76%) aged 25 to 34 years. Most respondents lived in rural settings (58.38%) and had attained secondary education (47.19%). Although educational disparities were evident, 29.02% of the respondents had no formal schooling, whereas only 7.76% of the respondents reached higher education. Household dynamics showed traditional patterns, with 73.18% of respondents coming from male-headed households and 68.04% of respondents being married. The economic vulnerability was apparent as 56.35% of respondents belonged to poor wealth quintiles, compounded by crowded living conditions (93.42% in households with ≥10 members) (Table 2).

**Table 2:** Sociodemographic and obstetrical characteristics of pregnant women in Ghana, 2022 Ghana Demographic and Health Survey (N=5075)

Variable	Categories	Weighted frequency (%)
Age (y)	15–24	1347 (26.48)
	25–34	2426 (47.76)
	35–49	1310 (25.75)
Residence	Urban	2113 (41.62)
	Rural	2970 (58.38)
Mother’s educational level	No education	1478 (29.02)
	Primary	814 (16.02)
	Secondary	2397 (47.19)
	Higher	394 (7.76)
Wealth index	Poor	1646 (56.35)
	Middle	761 (18.17)
	Rich	1510 (25.48)
Marital status	Married	3460 (68.04)
	Unmarried	1623 (31.96)
Parity	≤2	2396 (47.13)
	≥3	2687 (52.87)
Sex of the household head	Male	3722 (73.18)
	Female	1361 (26.82)
Household size	1–4	152 (3.00)
	5–9	182 (3.59)
	≥10	4749 (93.42)
Birth order	First	1320 (26.04)
	Second	1062 (20.94)
	Third	825 (16.28)
	Fourth	1862 (36.73)
Parental education	No education	1359 (31.00)
	Primary	511 (11.71)
	Secondary	1870 (42.83)
	Higher	632 (14.46)
Age of the household (y)	<35	1878 (36.99)
	35–44	1674 (32.89)
	45–54	738 (14.52)
	55–64	425 (8.35)
	>65	368 (7.25)
Media exposer	Yes	1506 (29.67)
	No	3569 (70.33)

### Reproductive profile and information access

Reproductive patterns revealed high parity: 52.87% of women had ≥3 children, and 36.73% of women were expecting their fourth or subsequent child. Concerning information access, only 29.67% of respondents had media exposure, indicating potential barriers to health communication. Intergenerational educational trends mirrored maternal patterns, with 31.00% of parents having no formal education and merely 14.46% of parents achieving higher education. Household leadership showed a younger age, with 69.88% of the household heads below the age of 45 years (Table 2).

### Prevalence of early antenatal care booking

This study found that 65.06% of pregnant women in Ghana attended ANC early (Figure).

**Figure:**
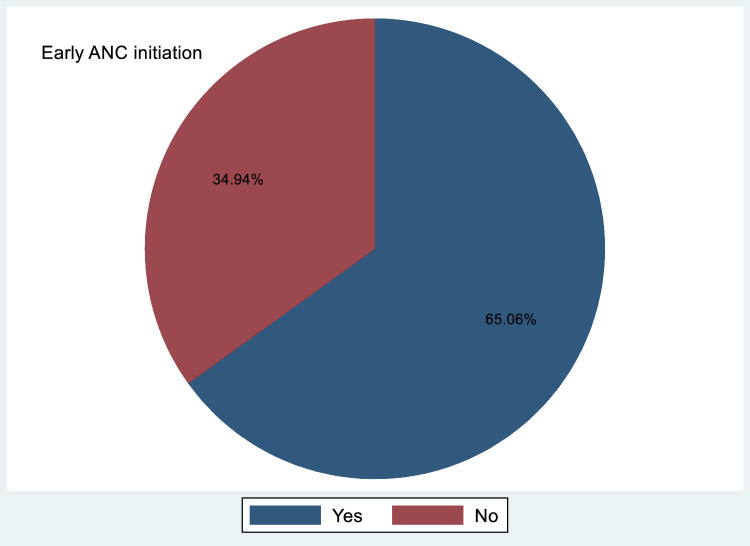
Prevalence of early ANC booking in Ghana, GDHS 2022 (N=5075) *ANC*, antenatal care; *GDHS*, Ghana Demographic and Health Survey.

Tilahun. Prevalence of early antenatal care visit in Ghana. Am J Obstet Gynecol Glob Rep 2026.

### Determinants of early antenatal care booking

After controlling for confounding variables, the adjusted odds ratios (AORs) revealed that older maternal age (25–49 years), higher education, and wealth remained significant predictors of early ANC attendance. Women aged 25 to 34 years (AOR, 1.38; *P*=.002) and 35 to 49 years (AOR, 1.62; *P*<.001) had higher odds of early ANC than younger women (15–24 years). Higher education (AOR, 2.53; *P*<.001) was the strongest predictor, urban residence (AOR, 1.87; *P*=.003), and wealthier women (AOR, 1.33; *P*=.010) showed increased odds. Conversely, higher parity (≥3 children; AOR, 0.59; *P*<.001) significantly reduced early ANC attendance, indicating that multiparous women were less likely to seek care early. These findings reaffirm the lasting effect of socioeconomic status (education and wealth) and maternal age on ANC use, highlighting the necessity for focused efforts to support younger, less-educated, and high-parity women in accessing early ANC (Table 3).

**Table 3:** Bivariate and multivariate analysis for determinants of early antenatal booking in Ghana, 2022 Ghana Demographic and Health Survey (N=5075)

Variables	Categories	Early ANC visit		COR (95% CI)	*P* value	AOR (95% CI)	*P* value
		Yes	No				
Age (y)	15–24	806	538	1	—	—	—
	25–34	1648	776	1.42 (1.23–1.63)	.009	1.38 (1.13–1.69)	.002
	35–49	848	459	1.23 (1.05–1.44)	.05	1.62 (1.25–2.11)	.000
Residence	Rural	1878	1085	—	—	—	—
	Urban	1424	688	1.19 (1.06–1.34)	.003	1.87 (1.95–2.82)	.003
Mother’s educational level	No education	895	578	—	—	—	—
	Primary	494	319	1.00 (0.84–1.19)	.999	0.97 (0.79–1.19)	.817
	Secondary	1574	821	1.24 (1.08–1.42)	.002	1.05 (0.87–1.27)	.555
	Higher	339	55	3.98 (2.94–5.39)	.000	2.53 (1.72–3.73)	.00
Wealth index	Poor	1738	1122	—	—	—	—
	Middle	601	321	1.20 (1.04–1.41)	.016	1.17 (0.96–1.44)	.111
	Rich	963	330	1.88 (1.63–2.18)	.00	1.33 (1.07–1.66)	.010
Marital status	Unmarried	1012	610	—	—	—	—
	Married	2290	1163	1.18 (1.05–1.34)	.006	1.07 (0.91–1.27)	.36
Parity	≤2	1601	791	—	—	—	—
	≥3	1701	982	0.85 (0.76–0.960)	.008	0.59 (0.46–0.756)	.00
Birth order	First	876	442	—	—	—	—
	Second	715	345	1.05 (0.88–1.24)	.61	1.00 (0.84–1.19)	.817
	Third	577	247	1.18 (0.97–1.42)	.08	1.24 (0.08–1.42)	.555
	Fourth	1124	735	0.77 (0.66–0.89)	.001	3.98 (0.94–2.39)	.75
Parental education	No education	815	538	—	—	—	—
	Primary	317	194	1.08 (0.87–1.33)	.478	1.00 (0.80–1.25)	.98
	Secondary	1274	595	1.41 (1.22–1.64)	.000	1.13 (0.94–1.35)	.18
	Higher	475	156	2.00 (1.63–2.48)	.000	0.96 (0.73–1.27)	.80
Age of the household	<35	1268	609	—	—	—	—
	35–44	1101	568	0.93 (0.80–1.07)	.317	0.94 (0.79–1.11)	.47
	45–54	437	300	0.69 (0.58–.834)	.000	0.79 (0.63–0.99)	.04
	55–64	265	159	0.80 (0.64–0.99)	.046	0.87 (0.67–1.14)	.34
	>65	231	137	0.80 (0.64–1.02)	.075	1.05 (0.78–1.41)	.72
Distance to health facility	Big problem	910	571	—	—	—	—
	Not a big problem	2392	1202	1.25 (1.10–1.41)	.001	1.11 (0.96–1.28)	.14
Media exposure	Yes	2267	1035	1.26 (1.11–1.43)	.00	1.07 (0.92–1.25)	.336
	No	471	1302	—	—	—	—

*ANC*, antenatal care; *AOR*, adjusted odds ratio; *CI*, confidence interval; *COR*, crude odds ratio.

## Discussion

Many health problems in pregnant women can be prevented, detected, and treated by trained health workers during ANC visits. The WHO recommends a minimum of 4 antenatal visits, composed of interventions, such as tetanus toxoid vaccination, screening and treatment of infections, and identification of warning signs during pregnancy. Such antenatal visits should be done in the first trimester of pregnancy (before 12 weeks of gestation).^32^^,^^33^ The identification of complications or risk factors for complications on such early visits enables early institution of interventions to alleviate or mitigate the effects of such complications on the mothers and unborn babies.^34^

This study analyzed the prevalence and determinants of early ANC booking using data from the 2022 GDHS. The findings revealed that 65.06% of pregnant women in Ghana attended their early ANC visit in 2022. Key determinants of early ANC booking included advanced maternal age (25–49 years), parity, higher educational attainment, and wealth index.

The study found that 65.06% of pregnant women in Ghana initiated early ANC visits, a rate consistent with research from Addis Ababa^20^ but notably higher than findings in Bahir Dar, Ethiopia (46.80%)^35^; Tigray, Ethiopia (27.50%)^36^; Sindh, Pakistan (48.00%)^37^; Nigeria (24.00%)^38^; Bangladesh (43.00%)^39^; and Tanzania (18.50%).^40^ However, Ghana’s prevalence was lower than that reported in Nepal.^21^ These discrepancies may stem from differences in healthcare accessibility, socioeconomic conditions, cultural norms, government initiatives, and urban-rural divides. Ghana’s progress in maternal healthcare, which is driven by policies, such as the National Health Insurance Scheme, which mitigates financial obstacles to ANC access, may partly explain its higher uptake than that of other regions.

The finding that older maternal age is associated with early ANC booking aligns with the study from Southern Nigeria,^14^^,^^41^^,^^42^ likely because older women have greater reproductive health experience and awareness of pregnancy risks, leading to prompt care-seeking. They often possess more financial stability and decision-making autonomy, enabling easier access to ANC services, and their previous interactions with healthcare systems increase familiarity and confidence in seeking care. In addition, older women may receive stronger social support and are more likely to have planned pregnancies, reinforcing early ANC attendance, whereas younger women, particularly adolescents, may face financial constraints, social stigma, or lack of autonomy, delaying ANC initiation.^43^ This consistent pattern across different settings underscores the influence of age-related biological, socioeconomic, and cultural factors on ANC use.

The findings of this study revealed that the residency of the mother is a determinant of early ANC booking. This finding is in line with the studies conducted in Southern Nigeria^14^; Debre Berhan, Ethiopia^44^; South Africa^24^; and Zambia.^45^ This consistency points to the fact that women from urban residences might have better access to healthcare facilities than those from rural areas. Women from urban areas may have had access to education and had media exposure, and distance from health facilities did not pose a substantial barrier to early booking of ANC.^46^ Furthermore, pregnant women from rural areas might have poor maternal healthcare utilization due to a lack of accessibility and availability of the service.^47^

The other important determinant of early ANC booking is educational level. Pregnant women who attend higher education book ANC earlier than those who have no education. This finding is in line with the studies conducted in Ambo, Central Ethiopia^48^; Southern Nigeria^14^; Nepal^49^; Tanzania^41^; Bangladesh^50^; Eastern Province, Zambia^51^; and Pakistan.^37^ The possible explanation might be that higher education correlates with socioeconomic empowerment, enabling individuals to overcome financial barriers, fostering autonomy to make independent healthcare decisions, and reducing reliance on familial or spousal approval.^52^ In addition, educated women can navigate healthcare systems more efficiently, advocate for their needs, and challenge cultural stigmas around early pregnancy disclosure, and their exposure to progressive norms and supportive networks further encourages timely ANC initiation. Thus, addressing educational disparities through health education and policies can mitigate inequities in maternal healthcare access and outcomes.

The findings of this study revealed that the wealth index of women is a determinant of early ANC booking. Women with a wealth index categorized as rich had early ANC booking compared with those with a wealth index categorized as poor or middle. This finding is consistent with those of other previous studies conducted in Ethiopia^53^^,^^54^; Ambo, Central Ethiopia^48^; Tanzania^41^; and Pakistan.^37^ Women from wealthier households might have better autonomy, better educational level, and confidence in using maternal healthcare utilization.^55^, ^56^, ^57^ Because of family-related workload or other obligations, poorer women are less likely to obtain permission from their husbands and family members to visit a health facility for an ANC booking. Furthermore, wealth is expected to have a positive relationship with ANC because the use of the service is associated with the cost of consultation and the purchase of recommended medication alongside other indirect costs, such as transportation costs. Thus, it is expected that the higher the wealth of the woman, the more likely she is to use ANC because she may be able to afford the cost and other expenses that come with using the service.

Higher parity was associated with late initiation of ANC visits in this study. Similar results have been reported,^58^, ^59^, ^60^, ^61^ likely because multiparous women, drawing from previous pregnancy experiences, may perceive less urgency to seek early care, whereas competing demands from caring for multiple children and household responsibilities could further delay ANC attendance, compounded by potential overconfidence in managing pregnancy without medical supervision and financial or logistical constraints that discourage timely visits.

The study’s strength is that it uses national representative data, making it generalizable to all Ghanaian pregnant women. Because the data were self-reported, it might have been influenced by recall bias.

### Conclusion

Approximately 65.06 % of pregnant women book their first ANC before 12 weeks of gestation. The key determinants of early ANC booking in Ghana included maternal age, geographic location (urban vs rural residence), educational attainment, and socioeconomic status (measured using the wealth index). To address these disparities, targeted interventions could focus on enhancing educational outreach, such as disseminating awareness campaigns through media platforms to emphasize the importance of timely ANC initiation, particularly in rural communities. Expanding health insurance coverage could further reduce financial barriers, ensuring equitable access to maternal services, such as early ANC. In addition, policymakers should prioritize economic empowerment initiatives, including poverty reduction programs and income-generating opportunities, to mitigate socioeconomic inequalities and improve healthcare access for women in lower wealth quintiles.

## CRediT authorship contribution statement

**Befkad Derese Tilahun:** Writing – review & editing, Visualization, Validation, Supervision, Software, Methodology, Investigation, Conceptualization. **Mulat Ayele:** Writing – review & editing, Writing – original draft, Visualization, Validation, Investigation, Formal analysis. **Eyob Shitie Lake:** Writing – review & editing, Supervision, Methodology, Investigation, Formal analysis, Conceptualization. **Bogale Molla:** Writing – review & editing, Writing – original draft, Validation, Supervision, Methodology, Investigation, Conceptualization. **Gizachew Yilak:** Writing – review & editing, Writing – original draft, Validation, Supervision, Software, Methodology, Investigation, Formal analysis. **Molla Azmeraw:** Writing – review & editing, Writing – original draft, Visualization, Validation, Project administration, Methodology, Formal analysis. **Tegene Atamenta Kitaw:** Writing – review & editing, Writing – original draft, Visualization, Validation, Supervision, Software, Data curation, Conceptualization.

## Glossary

ANC: Antenatal care
AOR: Adjusted odds ratio
CI: confidence interval
COR: Crude odds ratio
DHS: Demographic and Health Survey
EA: Enumeration area
GDHS: Ghana Demographic and Health Survey
MMR: Maternal mortality ratio
WHO: World Health Organization
